# The Personal Health Record Paradox: Health Care Professionals’ Perspectives and the Information Ecology of Personal Health Record Systems in Organizational and Clinical Settings

**DOI:** 10.2196/jmir.2443

**Published:** 2013-04-04

**Authors:** Kim M Nazi

**Affiliations:** ^1^Veterans and Consumers Health Informatics OfficeVeterans Health AdministrationDepartment of Veterans AffairsAlbany, NYUnited States

**Keywords:** personal health record (PHR), health professionals, attitudes, eHealth, electronic health records (EHRs), secure messaging, qualitative analysis

## Abstract

**Background:**

Despite significant consumer interest and anticipated benefits, overall adoption of personal health records (PHRs) remains relatively low. Understanding the consumer perspective is necessary, but insufficient by itself. Consumer PHR use also has broad implications for health care professionals and organizational delivery systems; however, these have received less attention. An exclusive focus on the PHR as a tool for consumer empowerment does not adequately take into account the social and organizational context of health care delivery, and the reciprocal nature of patient engagement.

**Objective:**

The purpose of this study was to examine the experiences of physicians, nurses, and pharmacists at the Department of Veterans Affairs (VA) using an organizationally sponsored PHR to develop insights into the interaction of technology and processes of health care delivery. The conceptual framework for the study draws on an information ecology perspective, which recognizes that a vibrant dynamic exists among technologies, people, practices, and values, accounting for both the values and norms of the participants and the practices of the local setting. The study explores the experiences and perspectives of VA health care professionals related to patient use of the My HealtheVet PHR portal and secure messaging systems.

**Methods:**

In-depth interviews were conducted with 30 VA health care professionals engaged in providing direct patient care who self-reported that they had experiences with at least 1 of 4 PHR features. Interviews were transcribed, coded, and analyzed to identify inductive themes. Organizational documents and artifacts were reviewed and analyzed to trace the trajectory of secure messaging implementation as part of the VA Patient Aligned Care Team (PACT) model.

**Results:**

Study findings revealed a variety of factors that have facilitated or inhibited PHR adoption, use, and endorsement of patient use by health care professionals. Health care professionals’ accounts and analysis of organizational documents revealed a multidimensional dynamic between the trajectory of secure messaging implementation and its impact on organizational actors and their use of technology, influencing workflow, practices, and the flow of information. In effect, secure messaging was the missing element of complex information ecology and its implementation acted as a catalyst for change. Secure messaging was found to have important consequences for access, communication, patient self-report, and patient/provider relationships.

**Conclusions:**

Study findings have direct implications for the development and implementation of PHR systems to ensure adequate training and support for health care professionals, alignment with clinical workflow, and features that enable information sharing and communication. Study findings highlight the importance of clinician endorsement and engagement, and the need to further examine both intended and unintended consequences of use. This research provides an integral step toward better understanding the social and organizational context and impact of PHR and secure messaging use in clinical practice settings.

## Introduction

Personal health records (PHRs) are designed as tools to engage patients in their health care and to enable them to manage their personal health information [1-6]. Significant investments have been made by organizations to offer PHRs based on the desire to enhance patient-centered care [7-11], and the perceived potential for health care system improvement [12,13]. Historically, most PHR implementation efforts have focused on broad conceptions of consumer empowerment in which advocates emphasize the potential for PHRs to (1) increase consumer access to and control over health information, and (2) enable active patient participation in health care decision making and health management [14,15]. Despite high consumer interest in PHRs [16-21] and growing availability [22-25], a paradox exists in that adoption remains relatively low overall [26-28]. A national consumer survey conducted by the Markle Foundation revealed that only 10% of American adults currently use a PHR [29]. One notable exception to the low rate of PHR adoption is Kaiser Permanente: at the end of 2012, 4 million of its 9 million members had registered to use its patient portal, My Health Manager [30]. Among veterans, 71% utilize the Internet, and approximately one-fifth report using the US Department of Veterans Affairs (VA) PHR, My HealtheVet [31].

Preliminary findings in the literature suggest that provider endorsement may be an important factor in a patient’s choice to adopt a PHR, and that continued clinician engagement with patient PHR use may be required to achieve and sustain anticipated positive outcomes [32,33]. A national consumer study of public use and attitudes toward PHRs concluded that doctors may hold the key to increasing adoption [28]. Although there has been a prominent focus on PHRs as tools to support consumers, the value that consumers derive from the use of a PHR will likely be directly affected by the attitudes and actions of health care providers and team members within the context of the clinical setting. What may be missing from the current consumer empowerment paradigm is a deeper understanding of how patient PHR use unfolds within the context of the health care interaction and how it influences the provision of services by health care professionals in organizational settings.

Although PHRs are designed as consumer-oriented tools, understanding the consumer perspective is necessary, but insufficient by itself. Consumer PHR use also has broad implications for health care providers and delivery systems; however, these have received less attention. Many authors agree that the social and organizational changes implicated in patient use of PHRs will require a significant culture change for medicine [34-38]. Others raise more practical issues for physician practices, such as ensuring appropriate safeguards for release of information to patients [39-42], and determining how best to manage potentially large volumes of self-reported information within the limited time allocated to the clinical visit [43].

Greenhalgh and Swinglehurst [44] present a strong case for studying technology use as a social practice calling for ethnographic approaches that recognize technologies both shape, and are shaped by, human action. Although a variety of anticipated benefits are attributed to the use of PHRs, there is a critical need to examine use from a social and organizational perspective. Given the persistent paradox between reported patient interest and anticipation of benefits with relatively low adoption and little evidence about impact, PHR use must be examined as a component of health care work, influenced by and influencing organizational actors and their work within the health care ecosystem. Without this understanding, the rhetoric of consumer empowerment may have minimal effects in everyday settings in which patients and health care professionals interact.

### Objective

The purpose of this study was to examine the experience of VA health care professionals whose patients use an organizationally sponsored PHR system (My HealtheVet) to develop insights into the interaction of technology and the processes of health care delivery. The study aimed to explore the experiences and perspectives of health care professionals related to patient use of 4 PHR features that could have important ramifications for health care professionals: patient health education resources, tools to support medication reconciliation, tools to enable patient tracking and self-reporting of data, and electronic communication via secure messaging.

### Setting

The Veterans Health Administration (VHA) of the VA is the largest integrated health care system in the United States with over 1600 sites of care. VA health care facilities provide a broad spectrum of medical, surgical, and rehabilitative care to approximately 5.5 million patients annually. The VHA was an early pioneer in utilizing an enterprise-wide electronic health record (EHR) system [45] and piloting a tethered PHR prototype [46]. The national My HealtheVet PHR portal [47] was released in November 2003.

The My HealtheVet portal enables veterans to create and maintain a Web-based PHR that provides access to patient health education information and resources, a comprehensive personal health journal, and electronic services, such as prescription refills and secure messaging [48-50]. All site visitors can access health education resources, and veterans who self-register for an account can create a customized PHR and request VA prescription refills. For veterans who are VA patients, a 1-time process of authentication enables access to selected data from the VA EHR, such as laboratory test results and medication history. The VA Blue Button was added to the portal in August 2010, enabling veterans to generate and download an electronic file that contains their personal health information [51].

My HealtheVet secure messaging enables authenticated users who are VA patients to interact with their health care providers and VA staff electronically to exchange nonurgent health-related information, to request an appointment or prescription renewal, or ask health-related questions. A triage process similar to the telephone triage process enables a member of the health care team to read and respond to incoming messages or assign action to another member of the team. Users can set their preferences to receive a notification via email that a new secure message is available. Messages that are not marked as completed in the system are escalated, generating an alert to health care team members. Members of the health care team can elect to save selected parts of the interaction as a progress note in the VA EHR.

Secure messaging was initially available for a limited number of participants to support alignment with clinical practice workflows and enable participant feedback to guide refinement of the application. With the VA transformation to the Patient Aligned Care Team (PACT) model [52], based on the medical home model, incremental expansion of secure messaging made the service available in primary care at all VA facilities in early 2011. Expansion to specialty and surgical care settings and nonclinical areas continues.

As of September 2012, the My HealtheVet PHR portal had served more than 1.9 million registrants, with more than 896,000 authenticated VA patients. The site had logged more than 76 million visits, and veterans had requested more than 33.5 million prescription refills. The VA Blue Button had been used by more than 636,000 unique users with more than 2.4 million file downloads. More than 479,000 VA patients had also opted-in to use secure messaging. Most veterans currently visit the site to use the pharmacy-related features and satisfaction with the site is high [9].

## Methods

Given the paucity of research about how patient use of PHRs is experienced by health care professionals in the social and organizational contexts in which they are situated, a qualitative methodology was deemed most appropriate because the goal of this study was to gain an understanding of actors’ experiences and perspectives [53-56]. The study consisted of conducting in-depth interviews with VA health care professionals to better understand their experiences and perspectives related to use of the organizationally sponsored PHR, My HealtheVet. Although the initial aim of the study was to focus on 4 specific PHR features that could have important ramifications for health care professionals (patient health education resources, tools to support medication reconciliation, tools to enable patient tracking and self-reporting of data, and electronic communication via secure messaging), secure messaging emerged as the prominent focus for the study because it was used most often by study participants. Given this shift in focus, an analysis of the organizational implementation of secure messaging and the parallel development and implementation of PACT was also undertaken to gain a deeper understanding of the adoption and use of this technology in an evolving situated context. The study was reviewed and approved by both the Washington DC VA Medical Center and the State University of New York at Albany Institutional Review Boards (IRBs).

### Sampling and Recruitment

The sampling strategy used for this study was purposeful and theoretical because of the variable level of adoption and uncertainty about the degree to which participants had experiences with patient PHR use [53]. The sample consisted of a stratified purposive sample of VHA health care professionals in 3 groups: health care providers (including physicians, physician assistants, nurse practitioners, and advance practice nurses), nurses, and pharmacists. Criteria for participation in the study included that the participant: (1) was a VHA staff member (health care provider, nurse, or pharmacist) involved in direct patient care, (2) reported having experience with patient use of 1 or more of 4 My HealtheVet features, and (3) was willing and available to participate in a 45 to 60 minute in-depth interview.

Participants were recruited directly at 3 VHA discipline-specific organizational meetings in which the target audience were health care professionals. Interested individuals who could self-identify as meeting the sampling criteria were invited to contact the research investigator. A second recruitment strategy consisted of snowball sampling using My HealtheVet coordinators as informants to identify potential study participants [57]. The My HealtheVet coordinator is an organizational role at each VA Medical Center and Veteran Integrated Service Network (VISN) tasked with leading local efforts to implement My HealtheVet. Similarly, additional referrals were received during the actual in-depth interviews. Theoretical saturation was reached with the completion of 30 interviews.

### Data Collection

Recruited participants consented in writing and were then asked to complete a short questionnaire that included general demographic questions and questions about their experience with patient use of the 4 My HealtheVet features of interest to validate study criteria and to enable a purposeful sampling of the participant pool. An interview guide was used for each interview, and the data collected through the short background questionnaire used to refine the focus of the individual interviews.

An initial round of 3 interviews (1 health care provider, 1 nurse, and 1 pharmacist) was conducted in January and February 2011 to pilot-test the interview guide and allow for refinements. Data collection was staged over time (February through July 2011) to enable iterative coding and analysis. Because study participants came from various locations across the country, interviews were conducted by telephone at a time that was convenient for the health care professional given their clinical schedule. All interviews were recorded using a digital audio recorder and were transcribed verbatim by the research investigator.

### Data Analysis

Responses to the short background questionnaires were recorded in a spreadsheet to facilitate analysis of the sample and continued purposive sampling. Review and analysis of the interview data was ongoing throughout the study in conjunction with continued data collection until theoretical saturation was reached [58]. The analysis process involved an ongoing review of all data and project documents to identify common themes utilizing modern techniques of qualitative analysis. Coding and analysis of the interview transcripts employed an inductive approach using the perceptions and reported experiences of participants as the basis for constructing and organizing the codes and categories [53]. Atlas Ti version 6.2 qualitative analysis software (Scientific Software Development, Berlin, Germany) was used to organize the interview transcripts, facilitate data coding and sorting, and document memos.

Interview transcripts were reviewed systematically using open coding [53,58-60] to develop an initial coding scheme. After an initial coding structure was developed, the data were reviewed iteratively as the study progressed to review and refine codes, and to identify additional codes. Analysis continued with axial coding to identify relationships between code categories. The constant comparison method [61] was used to continuously refine the codes and coding categories. After developing preliminary interpretations, all data were reviewed for possible alternative interpretations and rival conclusions. Field notes were prepared immediately following each interview and investigator memos were written to further document findings as they emerged from the data and analysis. Analytic memos captured emerging insights and connections between codes and themes, and integrative memos were used to develop theoretical connections between the coded data excerpts.

Based on the study findings generated by the analysis of the interview data, a review and analysis of organizational documents was also undertaken in October 2011 to reconstruct the history of secure messaging implementation and relevant organizational changes that happened in parallel. Documents that were included in this analysis consisted of workgroup meeting minutes, status reports, memorandums, statistical reports, and project implementation reports. Further analysis of the study participant characteristics based on their responses to the short background questionnaire coupled with data from their interview transcripts then enabled a deeper understanding of each participant and the context of their perspective in terms of their experience with respect to this trajectory.

Several analytic techniques were used to improve the validity and reliability of the study [62]. Study participants were assured of the anonymity of their comments. Other VA researchers with experience in qualitative research were consulted at key milestones in the project to review sample codes, memos, and themes. As the study progressed, participants in later interviews were asked at the end of their interview to comment on emerging themes from earlier participants. A summary of the implementation milestones and timeline was reviewed and validated by implementation leaders. Relevant reflections generated through reflexive processing were documented in a project journal and reviewed as part of the analytic process. Member checking was also performed by inviting all study participants to review a summary of findings to check the authenticity of the investigator’s interpretations [63].

## Results

A total of 30 VA health care professionals participated in the study (10 health care providers, 10 nurses, and 10 pharmacists). As shown in Table 1, study participants reported working predominantly in primary care settings and spending the majority of their work time (62% on average) on direct patient care activities. Four health care professionals reported working in specialty care (eg, audiology).

### Comparison of Health Care Professionals’ Perspectives

The design of this study was intended to allow for a comparison of perspectives for 3 different types of health care professional roles: health care providers, nurses, and pharmacists. Themes were consistent across the 3 professions. This consistency may reflect the unifying nature of an integrated delivery system, especially because the system as a whole has recently undergone a systemic transformation to the PACT model. Where variations did exist, they represented differences in areas of focus reflective of varying roles rather than disagreements. For example, although health care providers vocalized concerns about the lack of workflow fit for tools to support patient self-reported data, nurses focused more on patient motivation, and pharmacists focused on practical workload implications. All 3 groups consistently emphasized the barriers associated with lack of access to patient self-reported data, and expressed concerns about the potential for mismatched patient expectations. For secure messaging, health care providers emphasized improving the quality of the clinical visit, whereas nurses uniquely emphasized the importance of providing patients with information that they could refer back to later. Pharmacists emphasized the potential workload burden for secure messaging because many messages request prescription refills, and these are triaged and assigned to pharmacists for completion. These nuances reflect the varying work tasks that health care professionals perform. All 3 groups consistently emphasized the positive consequences of secure messaging.

**Table 1:** Study participant gender and self-reported type of work and activity.

Characteristics		Providers (n=10)	Nurses (n=10)	Pharmacists (n=10)	All groups (N=30)
**Gender, n**					
	Male	6	0	4	10
	Female	4	10	6	20
**Type of work setting, n**					
	Primary care	7	8	9	24
	Specialty care	2	1	1	4
	Both/other	1	1	0	2
**Type of work activities, %**					
	Direct patient care	55	53	78	62
	Administrative work	28	43	14	28
	Other	17	4	8	10

### Key Findings

Prominent themes emerging from the study were organized into 5 key findings as shown in Textbox 1.

#### Underutilization of My HealtheVet Personal Health Record Features

In general, health care professionals reported limited experiences with patient use (and their own use) of My HealtheVet health education resources, tools to support medication reconciliation, and tools to support patient self-reported data (with some exceptions), often using alternative tools and resources instead. Health care professionals identified several barriers to use of the My HealtheVet PHR features, and commented that these barriers have also limited their endorsement of patient use.

#### Factors Inhibiting My HealtheVet Personal Health Record Adoption and Use

Health care professional’s accounts provide evidence that the My HealtheVet portal has been conceptualized by many as a tool for patients and separate from the clinical encounter, with some notable exceptions. Several study participants indicated that they had not experienced patient use of the tools, nor advised patients to use the tools, viewing My HealtheVet as a self-service portal for patients.

Many health care professionals reported general awareness of My HealtheVet but limited familiarity with its features, with the exception of secure messaging. Health care professionals note that this lack of knowledge limits their ability to endorse patient use, or to integrate use of My HealtheVet features within the clinical practice setting. They emphasize that increasing staff knowledge about the various features would enable staff to better utilize the available tools and resources, and to encourage patient use. Many commented that time constraints hamper their ability to become more familiar with these resources, and also to educate patients about them.

To educate both staff and patients, health care professionals emphasize that demonstrating functionality is important, advocating for approaches that enable hands-on experiences. Several commented that providing staff with opportunities to learn more about My HealtheVet will require that time be allocated to these activities with sufficient coverage of their patient care responsibilities. Availability of patient-accessible computers in the clinic setting was identified as an important structural need that would enable patients to learn more about the tools and to make use of them in concert with their clinical visit.

Health care professionals often reported using alternative tools and resources. For example, although My HealtheVet provides a significant library of health education resources, health care professionals already use alternative resources, such as subscription-based software that is linked from within the primary clinical workflow system or resources retrieved from the Internet, with little incentive to change. Health care professionals reported that they increasingly use Internet resources easily found by search engines, and speculated that patients do as well. As one health care provider said: “Why not just Google?”

Several health care professionals commented about the need to enable a delegation feature that would provide veterans with the ability to share their personal health information. They noted that given the current My HealtheVet system capabilities, patient-accessible computers within the clinic setting are needed so that patients can access these tools in conjunction with their visit. Health care providers could then view and discuss patient-tracked data, nurses could demonstrate relevant patient health education materials, and pharmacists could review patient self-entered medication lists with the patients to update the medication list within the VA EHR. Some health care professionals noted that even with a delegation feature in the portal, logging into a system outside of the primary clinical workflow system would be a barrier for them.

Likewise, health care professionals indicated that the inability for patients to share information with their health care team through the My HealtheVet system limits the value of tools to support patient self-entered data. Although health care professionals report that tools to support patient-tracked health metrics, such as blood glucose readings, can be helpful, the lack of timely report and communication has made these tools less useful because delegation is not yet available. Health care professionals also expressed concerns that patients often believed that the health care team could view data that they had entered, further influencing health care professionals’ endorsement of use. Several noted that this lack of integration with the primary clinical information system deters use because self-entered data are inaccessible to the health care team.

Although My HealtheVet also provides tools to support medication reconciliation, health care professionals consistently reported that, regardless of whether patients supply their own medication lists, the process of medication reconciliation always comes down to communication. They consistently described medication reconciliation as a standardized process that inherently involves a dialog between the patient and members of the health care team, and often also involves the patient’s family member(s) or caregiver(s). One health care provider summarized this by saying that medication reconciliation is “a very complex animal” the goal of which is to “really figure out what the patient is taking.” Because the primary focus of the process is to update the medication list in the medical record, health care professionals begin and end the process with the medication list on record within the VA EHR, reviewing this list with the patient and providing the patient with a copy of the updated list. Although patient input is seen as essential to this process, health care professionals report that patient-supplied lists are often suspect, either because they are not up-to-date when brought to the periodic clinical visit or because they may contain other inaccuracies.

#### Implementation, Use, and Endorsement of Secure Messaging

In contrast to their experiences with other My HealtheVet features, health care professionals reported successfully using secure messaging and routinely endorsing patient use. Although several of the study participants were early adopters of secure messaging, others began use of the system as part of the organization-wide implementation of the PACT model. Analysis of the trajectory of implementation revealed that secure messaging began as an innovation project, spread to other sites via early adopters, and was ultimately assimilated and routinized throughout the system. A history of implementation milestones is shown in Table 2. Health care professionals’ accounts revealed several factors that have facilitated the adoption and use of secure messaging.

#### Factors Facilitating Secure Messaging Adoption and Use

Health care professionals’ accounts revealed that in contrast to other My HealtheVet features, secure messaging has been perceived by health care professionals as a tool that has significant value for both themselves and their patients, and as having attributes that encourage adoption, including relative advantage over existing alternatives, compatibility with existing clinical systems, and fit with existing workflow. Secure messaging was implemented in a way that allowed for organic growth in use of the system, with opportunities to try out the system with a small number of patients, observe the success of others, and interact with other users. As implementation progressed, it led to the emergence of structures that further facilitated adoption, including the development of performance measures, decentralization of patient authentication processes within local clinic settings, and the emergence of clinical reminders within the VA EHR to prompt endorsement at patient visits.

In contrast to the My HealtheVet PHR, secure messaging implementation was also accompanied by training and education programs for health care professionals. Part of this training was devoted to alignment of system use with the clinical workflow. With the organizational implementation of the PACT model, this training was integrated into the PACT curriculum, which was then systematically offered to VA health care team members across the country.

Health care professionals report that the secure messaging system has been fairly well aligned with clinical workflow and implementation teams have invested time in structuring their triage teams and associated tasks to optimize this alignment. Although they suggest ways in which the system could be further integrated into the primary clinical information system, the alignments that do exist (such as the automatic notification of new and escalated messages and the ability to save secure messaging interactions in the VA EHR) facilitate use of the system. Health care professional’s accounts provide evidence that secure messaging has also addressed some of the barriers that previously constrained use of My HealtheVet PHR features, for example, by enabling more timely self-reports from patients between clinical visits, supporting communication and feedback, and facilitating documentation updates in the VA EHR.

#### Reported Consequences of Secure Messaging Use

Health care professionals report several consequences as a result of secure messaging use. These are summarized in Table 3. Health care professionals perceive that secure messaging improves patient access to the health care team and health care services, and makes it easier for health care team members to respond directly to patients. Secure messaging enables better connectivity between patients and members of their health care team and avoids some of the challenges encountered with telephone calls, such as phone tag. Patient perceptions about access are positively influenced, increasing patient’s confidence that they can easily reach their health care provider when needed.

Health care professionals perceive that secure messaging changes both communication and the patterns of communication. Communication is more direct and focused, in contrast to telephone communication. Health care professionals report differences in the patterns of communication with secure messaging as a result of asynchronicity, including more frequent communication with patients between periodic in-person visits, and a lowering of the threshold at which patients will initiate communication with their health care team.

Asynchronicity is perceived as beneficial because it enables patients and health care professionals to send and respond to messages when it is convenient for them. This enables patients to communicate in their own time and be thoughtful about their needs. For staff, asynchronicity enables them to respond to patient requests when they have time within their workflow and to give patient requests more focused attention. Health care professionals report that being able to save the interaction as a progress note in the VA EHR ensures needed documentation and also allows them to capture the patient’s description in their own words.

With secure messaging, health care professionals report that more frequent communication enables them to keep track of what is going on with their patients between face-to-face visits, and they know their patients better as a result. Exchange of information before the clinical visit also enhances the quality of the visit. As 1 provider described, accomplishing administrative work in advance of the visit enables the health care provider to focus on the patient’s agenda at the visit:

It gives you a conversation that you might not have otherwise had, except that you see them once every 7 to 8 months or 9 months or a year, you now have this interjected conversation piece that’s going on that allows you to find out what their value system is, what their reasons are, what the barriers are, how is it that they’re able to be successful with this piece or that piece. And then you can launch a change talk about other issues that may be the underlying root cause of why they’ve never been successful in the first place...So it’s a different kind of information gathering journey I would say...I would say that it supports or strengthens the relationship: patient to provider. In the most simple sentence that I could provide I would say that it strengthens the relationship. It certainly builds trust...I would say that it affects them all to date in a very positive way...the face-to-face visits seem to have a better flow. I have the patient set an agenda when I first walk into the room rather than ‘What are you here for?’ I say ‘What would YOU like to accomplish in this visit?’ And if they’ve been secure messaging me, then we’ve taken care of a lot of their list that they want to take forward to the provider, and I usually know...and we launch from there in the direction that the patient really wants to travel.

Another phenomenon that health care professionals describe is the impact of secure messaging on the threshold of communication for patients, facilitating improved communication with patients. Health care professionals’ accounts provide evidence that the interrelated effects of these changes leads to improved relationships between patients and members of the health care team. Secure messaging is perceived to increase patient engagement, trust, and satisfaction:

I think people get to communicate without the intensity of a visit. They get to do it in their own time. They get to be more thoughtful...it’s just a slam dunk...for them!...I think there’s 2 things that have changed...1 is the care coordination. I had an email: ‘I went to the Emergency Room. I had chest pains.’ Well, you know, boy I’m going to make sure that person has an appointment...I’m going to ask the clinic facilitator to get the records...and so...so...the patient has made me a better doctor...because, what if they didn’t let me know?...Well in addition to what I said about improving access, improving coordination...increasing my knowledge of the patients...between visits...and learning more about your patients...But that whole paradox of...even though it’s a computer communication, I actually know the patients better...would they have called me and told me that? On the phone? I’m not sure! I think...I think patients are behaving differently because we’ve lowered the threshold to share the information. And so...and because of that, I get to be more diligent.

Health care professionals also expressed concerns about workload, especially as use of the system increases. They consistently noted that workload so far has been manageable because use of the system has grown organically. They caution that as use of the system increases it will be important to continue to enhance the system, especially for integration with the primary clinical workflow system.

**Key findings and related themes.**

1. My HealtheVet PHR features have been underutilized, with limited patient endorsement.

Health care professionals report limited experiences with patient use (and their own use) of patient health education resources, tools to support medication reconciliation, and tools to enable patient tracking and self-reporting of data (with some notable exceptions)
Endorsement of patient use has been limited

2. Several factors have inhibited the My HealtheVet PHR adoption, use, and endorsement of patient use.

Lack of knowledge
Lack of perceived relevance
Perceived lack of relative advantage
Time constraints
Lack of alignment with workflow (eg, lack of integration with the primary clinical information system)
Lack of alignment with structures (eg, lack of patient-accessible computers in the clinic setting)
Lack of alignment with processes (eg, barriers to information flow)

3. In contrast, secure messaging has been more readily implemented, used, and endorsed by health care professionals.

Health care professionals report successfully using secure messaging, and endorsing patient use
Analysis of the trajectory of secure messaging implementation reflects spread and significant growth in use

4. Several factors have facilitated secure messaging adoption, use, and endorsement of patient use by health care professionals.

Perceived relevance
Perceived relative advantage
Education and training opportunities
Integration with the existing technology used to accomplish work tasks
Alignment with workflow within the clinical setting
Incentives that affect intended users (eg, performance measures)
Access to information entered by patients
Asynchronous, bidirectional communication for collaborative work

5. Secure messaging has had dramatic consequences for communication, patterns of communication, and patient/provider relationships.

Improves access and patient perceptions of access
Avoidance of telephone tag
Communication is more direct and focused
Improves convenience and efficiency
More frequent communication between periodic in-person visits
Lowers the threshold at which patients will initiate communication
Improved patient engagement, satisfaction, and trust
Enhances patient/provider relationships
Concerns about workload implications with increased use

**Table 2:** Milestones in the history of secure messaging implementation.

Date	Milestone
MAR 2004	Workgroup established to develop strategy for secure messaging.
MAY 2006	Workgroup initiates design and development of the secure messaging.
NOV 2007	Secure messaging deployed at 3 early adopter sites for pilot testing.
DEC 2007	Clinical workflow and triage process documents developed and distributed.
JAN 2008	Three additional sites added to initial 3 early adopter sites.
JUN 2008	Secure messaging application undergoes formal functionality testing.
SEP 2008	National release of secure messaging application within the My HealtheVet portal. Secure messaging tab appears for authenticated VA patients.
OCT 2008	Workload code approved and activated to capture workload credit. Encounter form developed to capture secure messaging progress note in the VA EHR.
DEC 2008	Secure messaging in limited use at 12 facilities in 8 VA health care systems. Every network (VISN) is required to establish a local implementation team.
JAN 2009	Clinical adoption toolkit released to field to support local implementation.
FEB 2009	VA National Universal Task Force releases report recommending transformation initiatives including new models of care.
APR 2010	VA initiates 3-year plan to implement Patient Aligned Care Teams (PACT) in more than 900 VA primary care clinics. More than 700 VA patients opted-in and actively using secure messaging with 136 triage groups.
JUL 2010	My HealtheVet coordinator positions formalized with initiation of recruitment.
AUG 2010	Secure messaging becomes part of the formal Operating Plan for New Models of Care (PACT).
SEP 2010	VA National Leadership Board formalizes performance targets: use of secure messaging within primary care at a minimum of 1 medical center per VISN within 30 days, availability of secure messaging within primary care at all medical centers within 1 year (September 2011), 100% penetration of secure messaging in all primary care clinics by September 2012.
OCT 2010	Annual national performance measures for fiscal year 2011 include 3 secure messaging–related goals (increase authentication, increase patients opted-in for secure messaging, increase number of sites offering secure messaging). Secure messaging enhancements released.
MAY 2011	Secure messaging offered within primary care at all VA medical centers, meeting national target in advance of September 2011 deadline.
OCT 2011	Annual national performance measures for fiscal year 2012 include 100% secure messaging penetration in primary care by March 2012, implementation within specialty and surgical care by September 2012, and aggressive targets for in-person authentication.
NOV 2011	More than 60 facilities reach FY12 milestone goal of 100% secure messaging penetration rate in primary care in advance of September 2012 deadline. One VISN has 100% secure messaging penetration in primary care for all facilities in the VISN. More than 58,019 patients actively using secure messaging with 6613 triage groups.

**Table 3:** Consequences of secure messaging.

Theme	Description
Improving access and patient perceptions about access	Health care professionals report that secure messaging improves patient access and influences patient perceptions about access by enabling better connectivity with the health care team and avoiding some of the difficulties encountered with telephone calls.
More direct communication	Health care professionals report that secure messaging has enabled more direct communication by enabling patients to send questions directly to their health care team and allowing health care team members to respond directly to patient inquiries.
Changing communication patterns/asynchronicity	Health care professionals report that for many kinds of needs an asynchronous Secure message is a more effective way to support patient communication with the health care team.
Changing communication patterns/lowering the threshold	Health care professionals perceive that secure messaging lowers the threshold at which patients will initiate communication with their health care team.
Changing communication patterns/enhancing relationships	Health care professionals report that secure messaging has had a positive impact on patient/provider relationships. Health care professionals attribute this to the patient’s perception of greater and more direct access to their health care team, the patient’s perception of better responsiveness of the health care team to their needs leading to greater respect, trust, comfort, and appreciation, and increased frequency of communication.
Concerns about workload	Health care professional express some concerns about workload implications as use of the secure messaging system increases.

## Discussion

**Figure 1:**
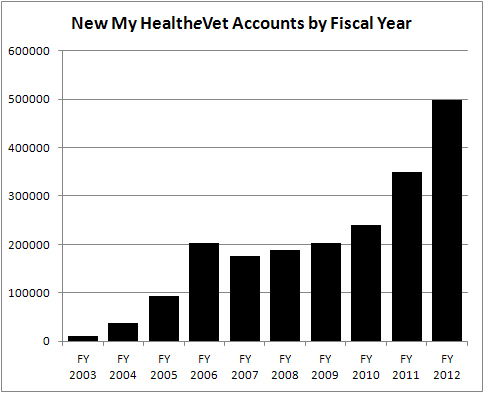
New My HealtheVet account registrations by fiscal year.

Study findings revealed that 3 My HealtheVet features (patient health education resources, tools to support medication reconciliation and tools to enable patient tracking, and self-reporting of data) have been generally underutilized, whereas secure messaging has been successfully implemented and used by health care professionals. Findings revealed several factors that have facilitated or inhibited the adoption, use, and endorsement of patient use by health care professionals. Health care professional’s accounts and analysis of organizational documents revealed a multidimensional dynamic between the trajectory of secure messaging and PACT model implementation and its impact on organizational actors and their use and endorsement of My HealtheVet. This dynamic has influenced workflow, work practices, communication, and the flow of information between patients and members of their health care team. In effect, secure messaging was the missing element of a complex information ecology and its implementation acted as a catalyst for change. Figure 1 illustrates the accelerated rate of growth in new My HealtheVet account registrations as secure messaging became more fully implemented. Secure messaging was also found to have dramatic consequences for communication, patterns of communication, and patient/provider relationships.

### Key Factors in the Implementation, Adoption, and Use of Technology

A comparison of the underutilized My HealtheVet features with use of secure messaging revealed 8 key factors that are important for the implementation, adoption, and use of a new technology in organizational settings (see Table 4).

### Perceived Relevance

Like other PHRs, My HealtheVet has generally been conceptualized as a set of tools for patients to utilize and as less relevant for health care professionals. Historically, promotional efforts have focused explicitly on patient use of the system with less attention to the potential relevance of these tools in the work of health care professionals. In contrast, secure messaging has been perceived by health care professionals as a tool that has significant relevance to their work. Promotional efforts have not only targeted patients, but have also focused on health care professionals to facilitate their adoption and use of the technology. As the organization has assimilated the PACT model, the relevance of secure messaging has been continually reinforced for professionals as an effective way to accomplish patient-centered care.

### Perceived Value

Study findings call attention to the question of value. Participants in this study recommended focusing on unique services that the My HealtheVet PHR offers, with great attention to secure messaging. For example, although My HealtheVet provides a significant library of patient health education resources, health care professionals already use alternative resources, with little incentive to change their practices. In contrast, secure messaging is perceived to offer specific advantages over existing alternatives. Many work tasks that health care professionals are responsible for require them to communicate with patients. Health care professionals report that secure messaging is more convenient than contacting patients by telephone, increases efficiency by avoiding telephone tag, and improves communication with patients by enabling increased communication between face-to-face visits. Each of these characteristics of secure messaging contributes to health care professional’s perceptions about its value, both for themselves and for their patients.

**Table 4:** Key factors in the implementation, adoption, and use of a new technology.

Key factor	Description
Perceived relevance	In order to be adopted, the new technology must first be perceived by individuals as relevant to their work.
Perceived value	In order to be adopted and used, a new technology that has been deemed relevant must then be perceived as having greater value than the available alternatives for accomplishing work tasks.
Education and training	In order to be adopted and used, the new technology must be implemented with education and training opportunities targeted toward the intended user to ensure that they have the knowledge and skills needed to make effective use of the technology.
Integration with existing technology	In order to be adopted and used, the new technology must be integrated with the existing technology that is being used to accomplish work tasks.
Alignment with workflow	In order to be adopted and used, the new technology must be aligned with the workflow within the particular setting of use.
Incentives	If the implementation of a new technology is accompanied by incentives that affect intended users, the adoption and use of the technology will be facilitated. Incentives can operate at the organizational level or at the individual and/or team level.
Access to information	If the new technology is intended to support the accomplishment of work tasks that are dependent upon access to information entered by patients, it must enable health care professionals to have easy access to that information.
Communication	If the new technology is intended to support collaborative work tasks involving multiple participants, it must support asynchronous and bidirectional communication in order to be adopted and used effectively.

### Education and Training

Since My HealtheVet was launched in 2003, education and training initiatives have primarily focused on veteran users. As a result, many health care professionals do not have adequate knowledge about specific features, and they have limited ability to make use of these features or to encourage and/or educate patients to make use of them. In contrast, secure messaging implementation has been accompanied by training and education programs for health care professionals, including opportunities for hands-on experiences. Training that was systematically being offered to VA health care team professionals across the country provided instruction on the use of the secure messaging system and also ways to align use of the system with the work tasks in a particular clinic, for example, by setting up the way that incoming secure messages are triaged and assigned for completion. In addition, health care professionals were also provided with ongoing opportunities to interact with other users, such as participation in weekly user-oriented conference calls. These forums help users to stay abreast of changes and updates to the system, and to learn from the experiences of their peers.

### Integration with Existing Technology

In the VA health care system, the technology used to accomplish and document the accomplishment of many work tasks is centralized within the VA EHR. The historical lack of integration of My HealtheVet tools with the VA EHR system has inhibited health care professionals’ adoption and endorsement of these features. In contrast, secure messaging has been purposefully integrated within existing technology systems. The ability to save secure messaging interactions directly from the secure messaging system into the VA EHR as a clinical progress note connects use of the new technology with the existing technology system to accomplish work tasks and to document related information. In addition to the VA EHR, health care professionals emphasize the value of automatic notifications for new and escalated secure messages via the enterprise-wide email system; however, they also recommend that these alerts and notifications be more fully integrated directly into the VA EHR.

### Alignment With Workflow

In organizational settings, workflow represents a commonly understood set of procedures for and sequence of work tasks, along with the assignment of specific roles for individuals to accomplish these work tasks. Taken together, these comprise processes that organizations manage to accomplish work. In health care settings, clinical workflow is a description of how the work is done and by whom. If technology is intended to be used to enable the accomplishment of specific work tasks, alignment with the larger workflow is also needed for its use to be effective and efficient for the health care team as an organizational work unit.

In contrast to other My HealtheVet features, secure messaging has been designed and implemented in ways that purposefully align with clinical workflow. As part of the process of secure messaging implementation, health care teams have invested time in structuring their triage teams to process secure messages in ways that are aligned with the procedures for, and sequence of, work tasks. One strategy that was used effectively early in the implementation of the system was to conduct a simulation exercise in which teams were gathered around a table, given a piece of paper to represent a secure message, and instructed to pass the paper to experience the triage process and identify how it would work most effectively. This exercise often revealed that having a nurse serve as the triage person would enable many requests to be handled without further assignment. Within some clinical teams, health care providers have chosen to receive and respond to incoming messages directly. This ability of the system to support local adaptation makes it adjustable to the procedures for and sequence of work tasks, taking into account the individual preferences of health care team members within a particular clinic setting.

### Incentives

Incentives can operate at the organizational level or at the individual and/or team level. Within the VA system, as with many organizations, performance measures are established each fiscal year, and progress is measured and monitored closely via enterprise-wide reports. At the individual level, incentives can include remuneration for work efforts that can be either financial (eg, reimbursement for activity) or nonfinancial (eg, workload credit for activity). In the VA system, attribution of workload credit is seen as an important factor because it influences the number of patients empaneled to a particular provider. In addition, health care providers are employed by the agency, and organizational factors, such as performance measures, exert significant influence (eg, pay for performance), especially in comparison to settings in which health care providers are not employed. Although historically VA performance measures have been predominantly focused on clinical quality measures, the addition of measures related to technology use exemplifies the use of incentives at the organizational level to facilitate the adoption and use of the technology.

The My HealtheVet system was launched in 2003, but organizational incentives for increased patient adoption of the system were not formally instituted across the VA system until secure messaging was added to the system. As VA concurrently transformed primary care settings to the PACT model, secure messaging use became part of this organization-wide initiative, ultimately leading to the development of national mandates for staff use and national performance goals to incentivize increased patient use. Because My HealtheVet account registration and user authentication are requirements for patients to adopt secure messaging, national performance measures also targeted increased patient authentication in the My HealtheVet system. Incentives drive the prioritization of staff activities, the allocation of resources, and the continuous measurement and monitoring of progress. At the individual level, a workload code for secure messaging has been activated to enable workload credit for secure messaging activity. Implementation efforts for a new technology should address the facilitating effects of incentives to foster increased adoption and use of the technology.

### Access to Information

The inability of patients to share information from within the My HealtheVet system has made these tools less useful for clinical care because information entered by patients is currently inaccessible to the health care team. In contrast, secure messaging has enabled patients to share information in a timely manner with their health care team. For example, management of diabetes often requires insulin titration based on a patient’s blood glucose readings. By using secure messaging, patients have been able to share their blood glucose readings with their health care team in an efficient and timely way, enabling health care team members to complete titration without a face-to-face visit.

### Communication

Communication is a crucial requirement for accomplishing collaborative work tasks. Many work activities in health care are collaborative in nature; for example, the process of medication reconciliation requires direct interaction between patients and health care professionals. Bidirectional communication supports not only the conveyance of information, but also the interaction between participants that is needed for validation, clarification, feedback, and ultimately the accomplishment of collaborative work tasks. Secure messaging has enabled patients to provide timely updates about changes in their medication usage, to correct any observed inaccuracies or omissions in their VA prescription history, and to enable the interactive dialog that is needed to ensure understanding and feedback in between periodic face-to-face visits. In this way, secure messaging supports the accomplishment of the process of medication reconciliation. Members of the health care team can then document these interactions in the VA EHR and make updates to the medication list on record that is used to make decisions about clinical care and treatment regimens.

### From Diffusion to Assimilation and Routinization

As predicted by diffusion of innovations theory [64], study findings demonstrate that having adequate knowledge of the technology and its features is a prerequisite for adoption and assimilation. Given the prevalent lack of knowledge about My HealtheVet features among health care professionals and the perception of it as a tool solely for patients, little attention has been given historically to engaging patients about use of the tools in clinical settings. Other studies have found that health care professionals generally have limited knowledge about PHRs [65,66] and a relatively narrow view of PHR functions [67]. Extensions of diffusion of innovations theory also place strong emphasis on other organizational and social factors. Greenhalgh and colleagues [68] caution that the perceived attributes of technology are neither stable features nor sure determinants of adoption or assimilation. Instead, it is the interaction among the technology, actor, and a particular context that determines adoption and use. As initial adoption evolves into assimilation and routinization, organizational and social factors are increasingly influential factors, especially for complex process-based innovations. Often the unit of assimilation in organizations is the team or department, exemplified in this study by the secure messaging triage team. For these reasons, they emphasize that although the standard attributes of diffusion of innovations theory are important and relevant, they are insufficient by themselves to explain the adoption and assimilation of complex innovations in organizations. Other factors, such as social and organizational influences, must also be examined.

### Implications for the Evolution of Personal Health Records

The growing body of literature about PHRs and secure messaging is beginning to demonstrate that to be most effective for patients and their health care providers, PHRs should be combined with Web-based messaging tools to support information sharing and bidirectional communication. Several studies have begun to emphasize the important role of communication with patients as they make use of PHRs [69-71]. As Terry notes [72], “a PHR that doesn’t connect to your doctor is like an ATM without any money in it.” Systems that provide patients with access to their laboratory test results, for example, should also anticipate the need for additional communication by providing patients with the ability to ask questions about their results to “close the loop.” These findings have important implications for the design and use of PHRs and PHR systems.

### Implications for Practitioners, Organizations, and Researchers

Study findings have important implications for individual practitioners, organizations, and researchers. These implications should be considered at the system level, the organizational level, and the individual level [48]. Findings in this study provide evidence that at the system level, the integration of secure messaging into the PACT model as a new model of care institutionalized use of the technology with a systemic implementation program, incentivizing performance measures, and an enterprise shift to patient-centered, team-based care. At the organizational level, integration within clinical practice settings has improved access, provided operational efficiencies, and enabled alignment of the technology within the clinical workflow. At the individual level, health care professionals report that secure messaging has improved patient engagement, and enhanced the relationships that patients have with their health care team. These findings suggest that secure messaging has promising potential to improve health care delivery by complementing traditional methods of communication. These findings also illustrate the complexity of implementing technology in health care organizations, and the need to examine the implementation and use of technology at multiple intersecting levels.

### The Personal Health Record Paradox: Looking Beyond Commonly Reported Barriers

Much of the literature about PHRs to date has been focused on an accounting of PHR features and consumer perspectives about their use. Although some authors speculate about the potential impact of use on medicine and the patient-provider relationship, less attention has been placed on understanding the actual experiences and perspectives of health care professionals with respect to use of PHRs and PHR systems. Given the persistent paradox between reported patient interest in PHRs and anticipation of benefits with relatively low adoption, this study examined PHR use within a particular organizational ecosystem as a component of health care work. Although the literature generally highlights concerns about privacy and security as prominent barriers to the adoption and use of PHRs, this study provided a unique opportunity to look beyond these commonly reported issues to enable a deeper understanding of how patient PHR use may unfold within the context of the health care interaction and impact the provision of services by health care professionals in an organizational setting.

The VA health care system is an opportune environment to study the situated use of PHR features because veteran users express confidence in the system [9], health care professionals have embedded experiences working in an electronically mediated environment [73], and health care professionals are directly employed by the system. As a result, study findings highlight 4 implications for health care systems beyond the commonly reported barriers of privacy and security. First, health care professionals play a crucial role in the endorsement of PHRs to patients, and in subsequent engagement with patient use of PHR tools. Second, in order for health care professionals to adopt and use PHRs effectively, there are several key factors that must be present, including adequate education and training opportunities. Third, for technology to be effective in supporting and improving health care delivery, careful attention must be paid to align use of the technology with health care processes including incumbent work activities, information flow, and bidirectional communication when the process requires collaborative work. Fourth, increased patient use of PHRs and secure messaging may have significant workload implications for health care professionals that will need to be addressed. These implications raise important issues for organizations seeking to use technology such as PHRs to improve patient care.

### Clinician Endorsement and Engagement

Study findings reveal the important influence of clinician endorsement and engagement in patient use of PHR tools. Although PHRs are designed as consumer-oriented tools intended to engage and empower patients, study findings suggest that engagement must be a reciprocal process. This reciprocity has been represented in the chronic care model as productive interactions between the “informed activated patient” and the “prepared proactive practice team” [74]. Similarly, the Care Transitions Intervention model highlights clinician engagement as an important component of effective PHR use [75], emphasizing the role of the health care provider and other members of the health care team in fostering effective patient use. Based on a national survey of physicians about PHRs, Wynia and colleagues [66] concluded that to derive optimal benefit from using PHRs, patients and physicians should use these tools together as partners. Dunbrack [76] similarly emphasized that clinician endorsement is a primary motivator for patients to use a PHR. This assessment also predicts that the spread of patient-centered medical home models (such as VA’s PACT model) are likely to encourage clinicians to recommend PHRs to their patients to better manage their health and wellness.

Wynia and Dunn [42] caution that patients and providers have mixed views about PHRs that are not yet informed by direct experience. In a recent random national survey of US physicians, they found that 62% of physician respondents reported no previous experience with using electronic PHRs, although 42% said that they were willing to try using one with their patients. Similarly, consumer’s knowledge about PHRs continues to be limited, with more than half of consumers surveyed in February 2011 reporting that they were not familiar with the concept of a PHR [76]. Interestingly, 3 out of 4 consumers reported that they would start to use a PHR under certain circumstances, with 37% indicating that a clinician recommendation to use a PHR would be a primary motivator for them. The implications for health care systems are that better engagement of health care professionals may be needed to fully realize many of the broadly anticipated potential benefits of patient PHR use.

### Education and Training for Health Care Professionals

To engage clinicians and foster the adoption and use of PHR features, greater knowledge about PHRs and familiarity with their features is clearly needed. Study findings emphasize the importance of developing educational activities and promotional efforts aimed at health care professionals, taking into account their learning preferences and time constraints in the work environment. A great deal can be learned from implementation science about the most efficacious ways to meet this important need for education and training [77]. Further research is needed to develop and test interventions that will improve knowledge about a PHR system and its features. As the system is enhanced, it will also be important to include the ongoing dissemination of new information to health care professionals as part of an overall communication plan, and local champions may play an important role in this. Several health care professionals in this study were unaware of important changes that had been made to the system. The framework to support the implementation of secure messaging proposed by Wakefield et al [78] highlights these aspects of implementation as key factors.

### Health Care Processes, Information Flow, and Bidirectional Communication

Study findings suggest that to further examine how PHR tools can be integrated into health care work, a more holistic examination of the processes that embody work tasks and associated information flow is needed. For processes that require collaborative work, bidirectional communication is crucial. For example, an examination of the process of medication reconciliation revealed that sequential work tasks managed by health care professionals include a review of the medical list on record, interactive dialog with the patient to validate and identify changes including updates and amendments, and updating the list on record as an authoritative source. Simply providing tools for patients to document their medication information in their PHR was ineffective until this task was aligned with the larger process. Alignment with the process of medication reconciliation required asynchronous electronic communication that enabled the timely flow of information between patients and members of their health care team via bidirectional communication.

Findings from this study highlight the critical nature of information flow and bidirectional communication with 4 related considerations. PHR systems should enable patients to share information effectively with their health care team via tools such as delegation or the ability to authorize the addition of patient self-entered data to the official medical record [79]. Bidirectional communication tools should be implemented in tandem with PHRs to support the interactive dialog that optimal use of PHRs requires [23]. Integration with clinical workflow is a crucial determinant of use for health care professionals [80,81]. As use of PHRs increases, careful attention must be taken to address the potential for information overload [42], the need for complete and accurate information [82], and the potential for unintended consequences [83].

In this study, the organizational changes that occurred made it possible to witness the catalyzing impact of secure messaging related to information flow and bidirectional communication. Beyond the VA system, early standalone PHR models that lacked the capacity for information sharing and 2-way communication have given way to patient portals that include the ability to share information and support for electronic communication between patients and their health care team. Models, such as Kaiser Permanente’s My Health Manager, that integrate PHRs or patient access to EHR data with communication via secure messaging are more effective because they address these needs. Nijland et al [70] caution us to apply technology in ways that foresee the patient’s need for continuous and personalized feedback, especially for patients who have a greater need for care. These may be the same patients who are most highly motivated to utilize a PHR because of their need to manage a plethora of information related to their condition and/or their care.

### Implications Related to Workload

Although health care professionals have had concerns about the impact of secure messaging on workload, study findings provide evidence that workload to date has been manageable. Similarly, other studies have emphasized clinician’s concerns about additional workload [5,84-86], whereas several studies have found these concerns to be unfounded [87-90]. Even if increased workload is balanced by workflow efficiency, as some studies suggest, remuneration of time devoted to these activities may be important, whether through financial reimbursement [91] or workload credit for panel management.

### Study Limitations

This study has several limitations. By design, this study focused on health care professionals within the VA as an integrated system. The degree to which findings are generalizable to other organizations and systems is an area that warrants further study. Study participants varied significantly in their role or position within the larger organizational structure, for example, whereas some health care professionals had a prominent role in the national scope of the system, others were in a rural community-based clinic. This presents challenges in characterizing “the organization” for individuals. Additional research is needed to that more closely examines role conception and organizational structure as contextualizing factors.

Another limitation is related to the degree to which study participants have practical experiences with use of the system. Although the aim of the study was to understand experiences with 4 specific features of the PHR, the scope of the study was inherently limited by the lack of adoption for some My HealtheVet features. Even with this constraint, however, a great deal was revealed about facilitating and inhibiting factors. The temporal changes in the organization also necessitating adding to the scope of the study as it became important to trace the trajectory of secure messaging implementation when it became evident that its availability had an important influence on other things.

Lastly, the potential for study participants to perceive the investigator as an advocate of the program could influence their willingness to report negative accounts. This potential bias was minimized by recruitment strategies that targeted health care professionals from across the country providing direct patient care in the field rather than relying on known sources. All study participants were also reminded at the beginning of their interview to be candid, and were assured that their perspectives would be reported with anonymity. The willingness of study participants to voice both positive and negative experiences instills confidence in the credibility of individual accounts. In-depth interviews were crucial in going beyond initial assumptions based on the background questionnaire. Member checking entailed dissemination of a summary of key findings to all participants in the study, inviting participants to clarify, elaborate, or amend. Feedback from participants further validated these findings. Final interpretation of specific findings should bear these potential sources of bias in mind.

### Areas for Future Research

Findings from this study point to a number of areas for future research. These areas can be generally categorized into 5 domains as shown in Table 5. These areas further expand the My HealtheVet PHR research agenda [10] and other calls for additional research about PHRs and their use [92]. Significant progress is being made within VA via a collaborative partnership with the VA eHealth Quality Enhancement Research Initiative (QUERI) Center established in 2010 [93], and a number of studies about My HealtheVet are currently underway. Although lack of evidence about the distinct effects of PHRs and other eHealth tools on health and other outcomes persists [94], this research will be more feasible as actual use increases.

**Table 5:** Areas for additional research.

Domain	Description
Adoption	Further identify facilitators and inhibitors to adoption and use at multiple levels (system, organizational, individual) taking into account the various roles of health care professionals
Implementation	Develop approaches grounded in implementation science to measure the efficacy of implementation strategies
Education	Design and test interventions that will improve health care professionals’ knowledge and familiarity with the system and its features
Information flow	Model information flow and map to health care processes and activities across the patient trajectory to identify optimal ways to apply technology
Communication	Apply communication theory to further examine the nuances of asynchronous electronic communication

### Conclusions

The impetus for this study was the desire to deconstruct the PHR paradox: despite significant consumer interest and anticipated benefits, adoption and use of PHRs remains low, with some notable exceptions. After a decade of PHRs being promoted as independent tools to support a broad notion of consumer empowerment, a deeper understanding of these tools in the context of the social and organizational delivery of health care is needed to understand this paradox. Although there is anecdotal evidence that PHRs can improve health care, many of the benefits are presumptive and require further evaluation. Indeed, endorsement and engagement of health care professionals may be essential to fully realize the anticipated benefits of PHRs. As study findings demonstrate, patient use of PHRs also has broad implications for health care professionals and organizational delivery systems.

Although PHRs have been designed as consumer-oriented tools, health care professionals play a crucial role in the endorsement of PHRs to patients, and in subsequent engagement with patients’ use of these systems. Patient engagement may be most effective as a reciprocal process. In order for health care professionals to adopt and support patient use of PHRs effectively, there are several key factors that must be present, including adequate education and training opportunities. In addition, for technology to be effective in supporting and improving health care delivery, careful attention must be paid to align use of the technology with health care processes and clinical workflow including incumbent work activities, information flow, and bidirectional communication when processes require collaborative work. These implications raise important issues for organizations seeking to support technologies such as PHRs to improve patient care.

Viewing PHR systems as an information ecology highlights the dynamic interplay among technologies, people, practices, and values [95]. This interplay, however, also occurs in the context of an ecosystem in which organizational and social factors influence technology use. Changes to the ecosystem, exemplified in this study by the implementation of secure messaging, occur along a trajectory with the adoption and use of technology followed by assimilation and routinization. Changes to the flow of information, exemplified in this study by the addition of secure messaging to the My HealtheVet portal, effectively alter the dynamic, recursively changing the ecosystem as a result.

Leveraging technology in new and transformative ways that are most meaningful for patients and health care professionals will require a more holistic approach to better understand the social and organizational context of technology use in clinical practice settings. Given the institutional context of most health care service delivery models, the broader notion of the organization setting as an ecosystem warrants further attention.

## Abbreviations

EHR: electronic health record
IRB: Institutional Review Board
PACT: Patient Aligned Care Team
PHR: personal health record
QUERI: Quality Enhancement Research Initiative
VA: Veterans Affairs
VHA: Veterans Health Administration
VISN: Veteran Integrated Service Network
